# Sexuality, Lung Cancer, and the Older Adult: An Unlikely Trio?

**DOI:** 10.6004/jadpro.2013.4.5.5

**Published:** 2013-09-01

**Authors:** Anna Cathy Williams, Karen Reckamp, Bonnie Freeman, Rupinder Sidhu, Marcia Grant

**Affiliations:** From City of Hope, Duarte, California

## Abstract

**Case Study**

Mrs. L. is a 60-year-old retired female teacher with stage IIIA squamous cell carcinoma of the lung, status postchemoradiation. She recently developed radiation pneumonitis, which was managed conservatively, and she did not require steroids. Mrs. L. has noted some progression of her underlying dyspnea. She is monitoring her oxygen saturation at home, and most of the time it is in the range of 94% to 96%. On one occasion only, her oxygen dropped to 88% and rapidly improved to the mid-90s. Her cough has improved for the past 4 to 6 weeks. She denies sputum production, congestion, or fever. Mrs. L. does not require a walker and uses a wheelchair only for long distances. She has occasional, slight dysphagia. A recent CT scan shows stable disease, and she is to return to the clinic in 2 months for restaging and possible further chemotherapy.

Mrs. L. and her husband have been married for 33 years, and they have been very close. Until recently, they have continued to be sexually active and very intimate with each other. Since Mrs. L.’s diagnosis, and during treatment, the couple have become extremely stressed and psychologically spent. The act of sexual intercourse has ceased, yet they have attempted to remain close and maintain open communication. In addition to Mrs. L.’s increasing dyspnea, she has also suffered a great deal of fatigue and depression, along with alopecia and vaginal atrophy, due to the chemotherapy and radiation treatments.

Both Mr. and Mrs. L. are very distressed over the change in their sexual lives. Mr. L. has mentioned that he now feels more like a "nursemaid" than a husband or lover. Mrs. L. has made concerted efforts to maintain intimacy with her husband, but her fatigue is profound. She has taken to sleeping in the living room, sitting up on the couch, as it relieves her dyspnea to some degree.

Talking about sexuality in health care has slowly increased in recent years, along with efforts to augment support accompanying the occurrence of a catastrophic health event. Although some progress has been made, discussion about sexuality and intimacy, particularly in older adults, has been sparse (Katz, 2005; Lindau et al., 2007). While it is generally assumed that sexuality diminishes with age, research studies suggest that sexual interest and activity lasts well into the eighth decade of life. It is reported that 57% of individuals aged 65 to 74, and 26% of adults aged 74 through 84 remain sexually active (Hartford Institute for Geriatric Nursing, 2012). Sexuality and intimacy are at the very core of what makes us human beings. They are integral parts of any healthy relationship for people of all ages (Kalra, Subramanyam, & Pinto, 2011).

Older adults make up the most rapidly growing segment of the population. It is projected that 60% of 65-year-olds are expected to suffer at least one comorbidity, for example, diabetes, arthritis, congestive heart failure, or dementia, by the year 2030 (HealthyPeople, 2012). Combining these comorbidities with a diagnosis of lung cancer provides a challenging and complex picture of why individuals with this diagnosis might be concerned about their sexuality or intimate activities. Given that most individuals are diagnosed with lung cancer after the age of 65, with a median age of 72 (National Cancer Institute [NCI], 2012), the target population for this article will focus on older patients between 65 and 84 years old who have been diagnosed with lung cancer and who are in a partnership.

The following is a review of the evidence regarding sexuality and intimacy in the older adult and its relationship to the care of the older patient with lung cancer. This evaluation will include working definitions, topics regarding lung cancer and sexual health, the framework and hierarchy of needs in older adults, assessment of sexual concerns, and the etiology of sexual interruption. In addition, a quality-of-life (QOL) model will be used to illustrate how sexuality impacts a patient’s normal intimate functioning. Further topics will include a focused assessment, proper medical management, implications for sexuality assessment in practice, discussion of Mrs. L.’s case study, and a general discussion.

## Working Definitions

Sex is defined as the biological act leading to intercourse, as well as intercourse itself. Sexual health, according to the Centers for Disease Control (CDC) and the World Health Organization (WHO, 2013), is a state of physical, emotional, mental, and social well-being along with the integration of the somatic, emotional, intellectual, and social aspects of the sexual being in ways that are positively enriching, enhancing personality, communication, and love. Each patient is unique and defines his or her own sexuality according to age, gender, attitudes, as well as religious and cultural principles (Southard & Keller, 2008).

Sexual dysfunction or malfunction refers to difficulties experienced by an individual or a couple during any stage of a normal sexual activity, including desire, preference, arousal, and orgasm. Sexual dysfunction can have a profound impact on an individual’s perceived quality of sexual life (Eden & Wylie, 2009).

Sexuality is a complex biopsychosocial and spiritual state of mind. It is multidimensional to time, place, and partners and encompasses economic political, cultural, ethical, and legal aspects of life (WHO, 2013). Sexuality is natural and healthy, with a wide range of normal sexual functioning defined by an individual and one’s partner. It involves more than sex, and sex involves more than intercourse; it encompasses sexual knowledge, beliefs, attitudes, values, and individual behaviors. It is part of our identity, orientation, personality, thoughts, and feelings (WHO, 2013). The American Cancer Society (ACS, 2011b) defines sexuality as all of the feelings and actions associated with loving someone: holding hands, special looks, hugging, kissing, etc. It is not just the act of sex; it is intimacy. Although pathologic response to sexuality is beyond the scope of this article, its existence is acknowledged.

Intimacy is the extensive and often confidential knowledge that partners have concerning each other. It is caring, affection, interdependence, and the strong effect one person has on the other: mutuality. With intimacy comes trust and the tendency to think of the "us" not just the "me" (Kydd & Rowett, 2006).

Physical intimacy is sensual proximity or touching. It is an act or reaction to an expression of feelings (such as close friendship, love, or sexual attraction) that people have for one another. Examples of physical intimacy include being inside someone’s personal space, holding hands, hugging, kissing, caressing, and engaging in sexual activity. The forms of physical intimacy include physical closeness, touching (especially tenderly), touching intimate parts, and penetration. It is possible to be physically intimate with someone without actually touching. For instance, sustained eye contact is considered a form of physical intimacy that is analogous to touching. When a person enters someone else’s personal space for the purpose of being intimate, physical intimacy exists, with or without actual physical contact. Most people desire at least occasional physical intimacy, which is a natural part of human sexuality.

Research has shown that physical intimacy has health benefits (Lindau, Surawska, Paice, & Baron, 2011). A hug or touch can result in the release of oxytocin, dopamine, and serotonin and a reduction in stress hormones. A lack of physical intimacy can lead to increased feelings of loneliness, isolation, and depression (Lindau et al., 2011).

Emotional intimacy is an aspect of interpersonal relationships that varies in intensity from one relationship to another and varies from one time to another, much like physical intimacy. Affect, emotion, and feeling may refer to different phenomena. Emotional intimacy can be expressed through both verbal and nonverbal communication. The degree of comfort, effectiveness, and mutual experience of closeness might indicate emotional intimacy between individuals. Intimate communication is both expressed (e.g., talking) and implied (e.g., friends sitting close on a park bench in silence). Emotional intimacy depends primarily on trust, as well as on the nature of the relationship and the culture in which it is observed. Depending on the background and conventions of the participants, emotional intimacy might involve disclosing thoughts, feelings, and emotions in order to reach an understanding; offering mutual support; or building a sense of community. Additionally, it might involve sharing a duty without commentary (Kydd & Rowett, 2006).

**Figure 1:**
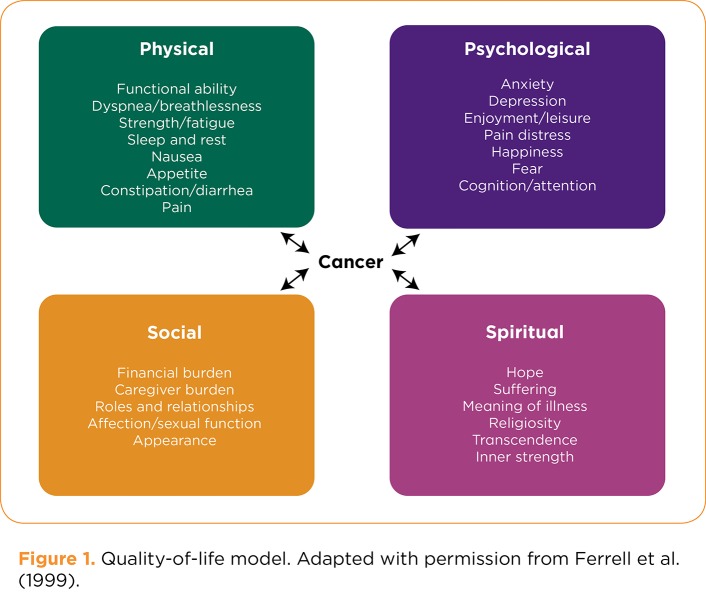
Figure 1. Quality-of-life model. Adapted with permission from Ferrell et al. (1999).

Quality of life is a broad concept that usually refers to a patient’s general well-being, including mental status, stress level, sexual function, and self-perceived health status (CDC, 2011a). Each person has a subjective definition of QOL. The City of Hope QOL Model (Ferrell et al., 1999) depicted in Figure 1 describes the physical, psychological, social, and spiritual aspects of a patient’s life.

Palliative care is an approach that improves the QOL of patients and their families facing the problems associated with a life-threatening illness, through the prevention and relief of suffering (WHO, 2002). Early identification, impeccable assessment, and effective treatment of pain and other problems (physical, psychosocial, and spiritual) are imperative. Palliation should include issues related to sexuality, which is often a silent QOL concern due to the intimate nature of the topic itself. Intimacy concerns frequently go unaddressed, leading to unmet needs and a negative impact on relationships and overall QOL.

## Lung Cancer and Sexual Health

Lung cancer takes approximately 160,000 lives annually, constituting 28% of all cancer deaths in the United States (CDC, 2011; Nwosu, Bayly, Gaunt, & Mayland, 2012). In the year 2009, there were approximately 388,000 men and women living with a diagnosis of lung cancer. This figure encompasses all those patients diagnosed at any point prior to January 1, 2009, including those with active disease and those deemed cured (NCI, 2012). Within a 5-year period, only 13.2% of those 388,000 individuals will still be alive (NCI, 2012).

Lung cancer remains one of the most medically challenging and burdensome diagnoses in all arenas of health care (NCI, 2013). Additionally, few studies have been conducted regarding the sexual health of older adults, let alone those also diagnosed with lung cancer. Prior to focusing on a review of intimacy associated with lung cancer, it is prudent to acknowledge the normal physiologic and pathologic changes that take place as a person ages. Individuals suffering from lung cancer may possess multiple comorbid conditions, along with numerous QOL issues. Frequently present at diagnosis are chronic illnesses including cardiac, pulmonary, endocrine, and psychological issues, in addition to common bodily alterations, for example, decreased libido, erectile dysfunction in men, and vaginal stenosis in women (National Institutes on Aging [NIA], 2012). Any one of these concerns can affect the ability to enjoy or participate in sexual activities or intimacy.

## Framework and Hierarchy of Needs

Considering the developmental stage of an elderly population (people aged 65 and older), Erikson’s theory of psychosocial development would place these individuals at the juncture of ego integrity vs. despair. Persons of this age group may experience a sense of integrity in having an accomplished life or may suffer despair at not having realized their goals (Erikson, 1950). Aside from Erikson’s framework of psychosocial stages, there have been relatively few theories of psychological development focusing on the later years. Sperry and Prosen (1996) have described aging as a developmental process and suggested that clinicians might better serve their patients by developing a more optimistic representation of aging. This would require a paradigm shift in the myths surrounding aging and the need to embrace the evidence surrounding the fact that aging is truly a developmental process.

**Figure 2:**
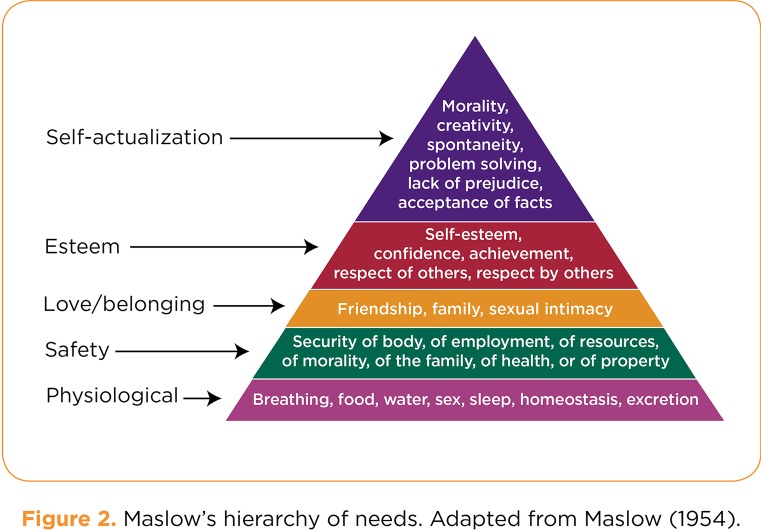
Figure 2. Maslow’s hierarchy of needs. Adapted from Maslow (1954).

Additionally, Maslow’s Hierarchy of Needs (1954), as seen in Figure 2, includes sex among the basic physiologic requirements, whereas sexual intimacy is elevated on the pyramid and is associated with love and belonging. Humans feel the need for partnerships and affectionate relationships as well as a sense of community. Viewed from a negative perspective, a lack of intimacy promotes vulnerability to loneliness and anxieties (Maslow, 1954). Sexuality signals closeness to another, boosting a sense of well-being, and should remain intact even in the presence of a life-threatening or terminal illness (ACS, 2011a).

## The City of Hope QOL Model

Ferrell et al. (1999) developed a functional model that includes four domains regarding QOL: physical, psychological, social, and spiritual well-being (Figure 1). The model provides a framework for examining the devastating effects lung cancer and its treatment have on a person’s ability to have interest in or engage in intimacy. Of a physical nature, the source of the issue could be the cancer treatment itself, with associated symptoms such as fatigue, nausea and vomiting, or the neuropathies of chemotherapy. Radiation often causes pneumonitis or fibrosis, while surgical resection can alter body image, interrupt tissue and nerve pathways, yield organ and hematologic toxicities, and diminish lung capacity. With preexisting issues, such as erectile dysfunction in men and stenosis or dyspareunia in women (Kagan, Holland, & Chalian, 2008; Rosenbaum & Rosenbaum, 2005), the diagnosis of lung cancer and/or tumor burden may further interrupt a person’s sexual well-being. Often accompanied by a chronic obstructive or restrictive condition, breathlessness often weakens one’s ability to perform sexually. It is reported that sexual dysfunction occurs in 20% to 100% of all patients with lung cancer (ACS, 2011a).

Psychological issues consist of a myriad of responses to having a diagnosis of cancer, along with a decrease in libido. Grief, anxiety, depression, loss of body image, feeling detached from self and others, and loss of control all contribute to fear and take a toll on interest in the sexual arena (Ferrell, Koczywas, Grannis, & Harrington, 2011).

Socially, many relationships may already be strained prior to a devastating diagnosis such as lung cancer. The effects of cancer treatment on finances and relationship roles can put any union at risk, causing a breakdown in communication, increased isolation, decreased affection, and increased fear of abandonment (Ferrell et al., 2011). These feelings can further intensify any issues of depression and anxiety that may accompany the cancer diagnosis.

Spiritual aspects, such as grieving and harm brought on by a life-threatening disease, are often ignored. An individual will struggle to find meaning and purpose in the experience and may question faith or spiritual beliefs. The suffering experienced, along with the uncertainties of the cancer trajectory, most assuredly impacts an individual’s sense of sexuality (Borneman, Ferrell, & Puchalski, 2010).

## Assessment of Sexual Concerns

The literature on sexuality issues in cancer patients is scarce at best. A study from the University of Chicago (Laumann, Das, & Waite, 2008) revealed that clinicians are failing to take into account any additional life domains aside from physical issues. The research also points to the fact that sexual problems are not an inevitable part of aging, but an element involved in the responses to the incidence of stressors in multiple life domains. It is evident from such data that health-care professionals should adopt a proactive psychosocial, sexual health, and satisfaction assessment as a standard intervention in health-care practice.

Further research by Basson et al. (2010) suggests that multifactorial issues in sexual health should be assessed and treated in a multidisciplinary fashion, with an emphasis on a structured interview involving both partners. According to Basson and colleagues, both direct and indirect factors should be evaluated, such as side effects from treatment or medication (direct) and diminished body image, change in mood, level of fatigue, and general relationship issues (indirect).

## Etiology of the Interruption of Sexuality With Lung Cancer

Although the manner in which a lung cancer patient demonstrates intimacy might change over time, there are numerous resources and medical interventions to address sexuality issues, all of which should be part of a comprehensive and dynamic assessment and care. Research by Lindau et al. (2007) revealed that although sexual activity declines with age, those engaged in intimate relationships viewed sexuality as an important aspect of life. However, 25% of older adults who remained sexually active admitted to refraining from activity due to health problems, such as those associated with advancing age and illness (Lindau et al., 2007). Additionally, patients with cancer admitted to both short- and long-term effects of their disease or treatment itself, curtailing their sexual function. Among the most common issues women reported were lack of desire, inability to reach orgasm, and atrophic vaginitis and stenosis. Men stated concerns surrounding erectile dysfunction, with only 14% of those utilizing pharmacologic intervention for the issue. Moreover, more men than women found treatment for sexual dysfunction necessary and/or satisfying (Lindau et al., 2007). Normal aging, chronic diseases, pharmacology, and the cancer itself can certainly diminish the sexual spirit (the manner in which one expresses sexuality).

Symptoms such as dyspnea coupled with erectile dysfunction or dyspareunia yields to an understanding of how intimacy is disrupted in relationships of the elderly. Etiology of the interruption of sexuality in lung cancer patients requires a thorough understanding of the personal, generational, and cultural influences shaping one’s sexual expression (Kagan, Holland, & Chalian, 2008). Although there is sparse evidence implying that early intervention prevents sexual morbidities, research does suggest that attention to the social (intimate) functioning of patients and their loved ones improves overall QOL, assisting in the basic physical and psychological functioning of any human being (Kalra, Subramanyam, & Pinto, 2011).

## Focus on Assessment

**Figure 3:**
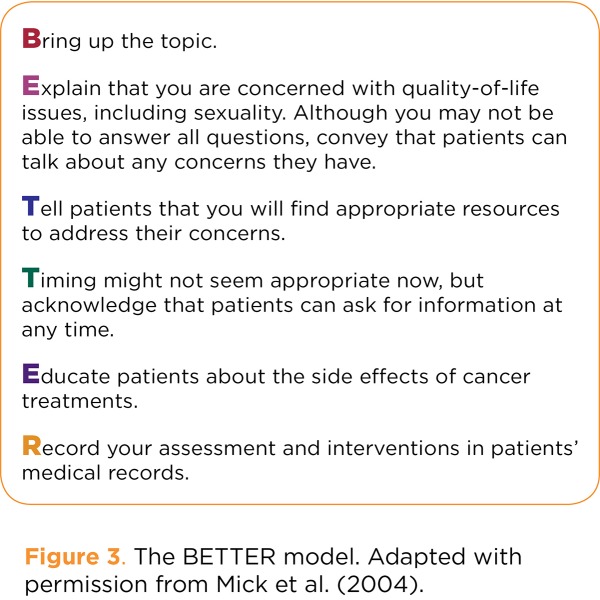
Figure 3. The BETTER model. Adapted with permission from Mick et al. (2004).

Common tools developed for sexuality assessment are the PLISSIT and BETTER models. The PLISSIT model suggests that the advanced practitioner follow these steps: Ask the patient for Permission to approach the subject of sexuality; offer Limited Information needed to function sexually; give Specific Suggestions for the individual to proceed with intimate relations; and provide Intensive Therapy surrounding the issues of sexuality for the patient (Annon, 1976). The BETTER model (Figure 3) proposes another approach for the advanced practitioner to assess sexuality in patients.

**Figure 4:**
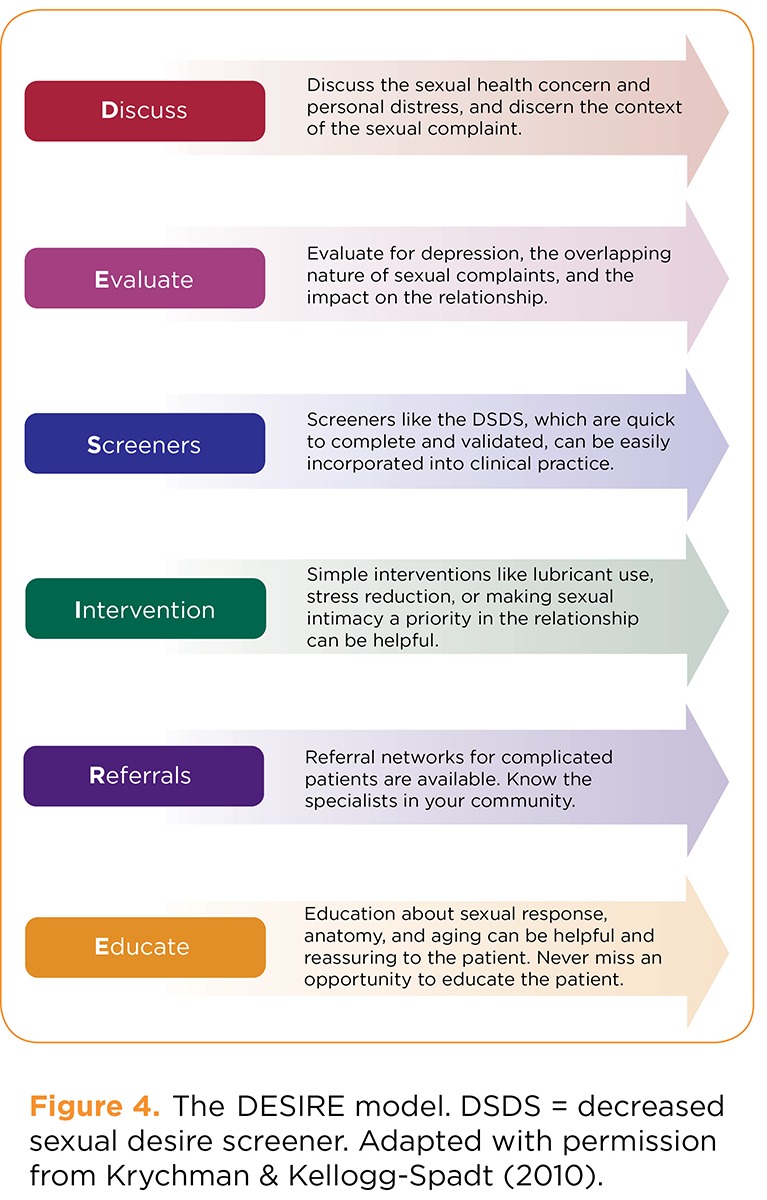
Figure 4. The DESIRE model. DSDS = decreased sexual desire screener. Adapted with permission from Krychman & Kellogg-Spadt (2010).

Another more contemporary outlook, the DESIRE model (Figure 4), lends a comprehensive approach to sexual assessment of the patient with cancer. Developed by Krychman and Kellogg-Spadt (2010), this approach takes into account the need for sexuality assessment, the complexities of sexual relationships, and the concerns related to sexual dysfunction associated with having cancer. They reported that although 20% to 100% of cancer patients cite sexual issues and concerns, a mere 13% are provided sexual assessment by the health-care team before treatment, and only 5% after treatment begins. It is estimated that 85% of adults would like to discuss sexual functioning with their physician; however, they do not for many reasons: 71% of patients believe their physician would not want or have the time to deal with sexual problems, 68% of adults are concerned about embarrassing their physician, and 76% believe no treatment is available for their problems. Krychman and Kellogg-Spadt encourage advanced practitioners to question patients regarding intimacy issues. As a clinician, one cannot treat a concern if its existence is not acknowledged (Krychman & Kellogg-Spadt, 2010).

## Medical Management of Sexuality Issues in Lung Cancer

Treatment of sexual issues is a multidimensional task that should be implemented as a collaborative effort. Interventions should be focused on both physical and psychological components, as it is difficult to feel sexual when in pain, chronically fatigued, or suffering from anxiety over a life-threatening illness. Both pharmacologic and nonpharmacologic resources may be employed. The use of supportive measures and community resources is essential.

Although initial prevention of intimacy concerns would be ideal, a diagnosis of lung cancer in an elderly patient ensures a complicated path. It does not, however, dictate that further problems are unavoidable. Management of sexual concerns should begin with a comprehensive evaluation of the four QOL domains (Figure 1). Next, a sexual assessment should be conducted using one of the previously described methods. This step will provide the information needed for a dynamic plan of care that is patient- and partner-centered. All interventions should focus on enhancing posttraumatic growth and maintaining or increasing communication between the patient and their significant other. Timely referrals to supportive team members should include consultations addressing the biopsychosocial, spiritual, and cultural aspects of care.

Symptom management of sexual issues may involve treating comorbidities due to the aging process, the cancer, or the treatments received. For example, as discussed under etiology, the plethora of issues brought about by the cancer or treatment itself can cause a multitude of physical maladies. Types of interventions for women might consist of using a water-based lubricant, which helps prevent tearing or damage to friable vaginal tissue (e.g., Astroglide, Replens, or Vagisil, which draw moisture to the area). Topical estrogen provides direct relief and limits overexposure of the hormone in the bloodstream. Practitioners may also weigh the benefits of "the ring" (estradiol vaginal ring) or an estrogen tablet for systemic relief of symptoms. Dilators are useful in maintaining the elasticity of the vagina, and the use of various pharmacologic agents has shown improved outcomes (Basson et al., 2010; van Lankveld et al., 2010).

For men, testosterone replacement, a phosphodiesterase inhibitor such as sildenafil citrate, or vacuum constriction devices generally achieve some level of success in generating and sustaining an erection. Self-injection (alprostadil [Trimix]) or urethral suppository therapy (alprostadil [MUSE]) relaxes the smooth muscle, promoting blood flow. Various remedies to be offered in the near future provide rapidly effective results, such as Uprima, which stimulates dopamine and dissolves under the tongue. A topical cream under investigation, Topiglan, acts in the same manner as alprostadil. Others, such as melanocortin, act on the central nervous system, and gene therapy produces proteins that assist in erectile function (WebMD, 2011). As with women, increased foreplay is an integral part of intimacy that promotes closeness of the couple. Penile implants or different sexual positions for promoting comfort for both partners may be sufficient. More challenging are interventions with those patients suffering from dyspnea and/or pain, unfortunately common in lung cancer. Again, a change in position may provide some satisfaction during intimacy, compensating for pain or dyspnea (ACS, 2011a; Schwartz & Plawecki, 2002; Hellstrom et al., 2010).

Sensate focus is a well-received intervention for sexual rehabilitation, relieving performance anxiety through the practice of exercises in intimacy. The concentration is on erogenous zones throughout a series of sessions (Van Hasselt & Hersen, 1996). Referrals for professional counseling and treatment programs directed at sexual function should be made early on in the cancer trajectory. Unfortunately, as research demonstrates, most consults for sexual concerns are instituted only when the patient begins to decline physically (Nwosu et al., 2012).

## Implications for Sexuality Assessment in Practice

Patient teaching should promote the desired outcomes of partners expressing feelings and communicating with one another. The couple should be able to identify potential and/or actual alterations in sexuality caused by the cancer or its treatment. Lastly, both partners should be able to describe strategies or alternate methods to use in response to sexual changes. Again, appropriate and timely referrals and information on additional resources are key in the care of lung cancer patients and their intimacy concerns (ACS, 2011a).

**CASE STUDY: FOLLOW-UP** 

In developing a plan of care for Mrs. L., we might assess intimacy with the BETTER model (Figure 3) and employ the QOL model (Figure 1) as well as a more comprehensive assessment. Physically, Mrs. L. is managed with respiratory medications and treatments and was referred to the hospital pulmonary rehabilitation program. She has been instructed to take two short naps daily and prioritize activities to preserve her energy. She is using vaginal dilators and Replens for her vaginal atrophy and dryness. She has also purchased three wigs and several beautiful headscarves and is wearing mascara and lipstick once again.

Psychologically, Mrs. L. was referred to clinical social work for an evaluation and was subsequently sent to a local psychiatrist for consultation and possible antidepressant therapy. Mr. L. has been asked to attend every other psychiatric session and is also being seen separately. Socially, due to Mr. L.’s concern of living in a two-story dwelling, the couple moved into a single-story residence in a senior community, where there are a multitude of local activities for both to attend. Mrs. L. attends aquatherapy classes three times a week, and Mr. L. plays golf almost daily. For her spiritual well-being, Mrs. L. was referred to the medical center chaplain; both Mr. and Mrs. L. have been in close contact with the chaplain.

Mr. and Mrs. L. have not been engaging in actual intercourse, yet they remain intimate on a very profound level. Their relationship includes sensate focus, utilizing a great deal of hugging, kissing, and caressing. Mrs. L. states that she feels ready to engage in sexual intercourse, which will require a position where she employs little movement, thus decreasing her dyspnea. Mr. L. is willing to try this process, and they are both very excited about the prospect of having "tangible" sex again.

Thus, in applying the BETTER model to this case study, it would be imperative to have Brought the topic up for discussion, Explained that any interventions were geared toward maintaining QOL, Told Mr. and Mrs. L. that appropriate resources would be provided and referrals made, along with reminding them that Timing is very important when planning care. Education should have been provided on a continuous and as-needed basis, with stellar Records of any plan of care and intervention maintained and updated routinely.

## Discussion

Far greater than the physiological act, sexuality and intimacy relate to our feelings and values, as well as our thoughts, desires, and experiences. A matter open to interpretation, our sexual selves involve many different dimensions in relationships, sexual or otherwise (WHO, 2013).

Care of an aging population includes a focus on the whole person: comorbid conditions, cancer diagnosis, and QOL concerns. Beyond the young and robust, older people and those with serious illness are interested in sexuality, yet intercourse is not the only avenue to pleasure. Sexuality should be a routine assessment, and communication should begin early on. If possible, assessment should be initiated before treatment begins in order to appropriately evaluate any alterations from the norm of the couple, as a sound baseline is the best reference point (Yi & Syrjala, 2009). Not only is it essential that the practitioner-patient discussion be timely, but the sexual partner should be involved in sexual assessment also, as he or she may have different responses concerning any sexual changes (Arena & Wallace, 2008; Yi & Syrjala, 2009). This discussion will open the door to an enhanced understanding of sexuality and intimacy while navigating a catastrophic disease such as lung cancer. Communication will allow incorporation of this understanding into the patient’s perception as a sexual being, dispelling misconceptions, judgment, and ultimately improving QOL for the patient and his or her partner.
